# THE EFFECT OF SHORT DURATION BED REST AND TYPE 2 DIABETES MELLITUS ON ARTERIAL STRUCTURE AND FUNCTION IN OLDER ADULTS

**DOI:** 10.1093/geroni/igac059.2530

**Published:** 2022-12-20

**Authors:** Kseniya Masterova, Tatiana Moro, Elena Volpi

**Affiliations:** University of Texas Medical Branch at Galveston, Houston, Texas, United States; University of Padua, Padova, Veneto, Italy; University of Texas Medical Branch, Galveston, Texas, United States

## Abstract

Type 2 diabetes mellitus (T2DM) and inactivity individually accelerate changes related to vascular aging. These changes increase cardiovascular risk and contribute to morbidity and mortality in the elderly. It is unknown if T2DM and bed rest have an additive, deleterious effect on vascular structure and function in older adults. The objective of this study is to determine the magnitude of the effect of bed rest on vascular structure in older adults with T2DM compared to healthy controls and determine if resistance exercise is protective of this effect. So far, we have recruited T2DM (n=9) and healthy control (n=17) subjects (age: 67.0 ± 6.7 years) to undergo five days of bed rest. During bed rest, subjects were randomized to receive intensive bedside resistance exercise physical therapy or standard of care in-bed passive physical therapy. On bed rest days 1 and 5, popliteal artery diameter, area, blood velocity, and blood flow were measured using Doppler ultrasonography. Our preliminary data show that the T2DM, non-exercise group had significantly greater decreases in popliteal artery diameter (-0.59 mm ± 0.18) than the control, non-exercise group (-0.17 mm ± 0.11). Resistance exercise did not prevent artery size changes in either controls or T2DM. Resistance exercise showed a trend in preventing blood velocity and flow reduction in both T2DM and control groups. These preliminary data suggest that older adults with T2DM had greater structural popliteal artery changes compared to controls. Resistance exercise appears to help maintain blood flow by maintaining/increasing popliteal artery blood velocity, but not size.

